# Numerical Prediction of the Mechanical Failure of the Intervertebral Disc under Complex Loading Conditions

**DOI:** 10.3390/ma10010031

**Published:** 2017-01-03

**Authors:** Gloria Casaroli, Tomaso Villa, Tito Bassani, Nikolaus Berger-Roscher, Hans-Joachim Wilke, Fabio Galbusera

**Affiliations:** 1 Laboratory of Biological Structure Mechanics (LaBS), Department of Chemistry, Materials and Chemical Engineering “Giulio Natta”, Politecnico di Milano, 20133 Milan, Italy; tomaso.villa@polimi.it; 2 IRCCS Istituto Ortopedico Galeazzi, 20161 Milan, Italy; tito.bassani@grupposandonato.it (T.B.); fabio.galbusera@grupposandonato.it (F.G.); 3 Institute of Orthopedic Research and Biomechanics, Trauma Research Center Ulm (ZTF), Ulm University, D-89081 Ulm, Germany; nikolaus.berger-roscher@uni-ulm.de (N.B.-R.); hans-joachim.wilke@uni-ulm.de (H.-J.W.)

**Keywords:** finite element analysis, intervertebral disc, ovine model, herniation, annulus fibrosus, anisotropic hyperelastic

## Abstract

Finite element modeling has been widely used to simulate the mechanical behavior of the intervertebral disc. Previous models have been generally limited to the prediction of the disc behavior under simple loading conditions, thus neglecting its response to complex loads, which may induce its failure. The aim of this study was to generate a finite element model of the ovine lumbar intervertebral disc, in which the annulus was characterized by an anisotropic hyperelastic formulation, and to use it to define which mechanical condition was unsafe for the disc. Based on published in vitro results, numerical analyses under combined flexion, lateral bending, and axial rotation with a magnitude double that of the physiological ones were performed. The simulations showed that flexion was the most unsafe load and an axial tensile stress greater than 10 MPa can cause disc failure. The numerical model here presented can be used to predict the failure of the disc under all loading conditions, which may support indications about the degree of safety of specific motions and daily activities, such as weight lifting.

## 1. Introduction

The mechanical causes of intervertebral disc (IVD) failures are still partially not understood. In recent decades, many research groups have studied the mechanical behavior of the intervertebral disc using different experimental set-ups and finite element models, obtaining conflicting results.

Many biomechanical studies have investigated the response of the functional spinal unit under specific loading conditions, in order to understand which loads or degree of bending were responsible for the disc failure [1,2,3], and if the degree of degeneration was related to the risk of generating structural failures [4,5,6]. The early studies investigated the effect of high compressive loads in combination with axial torsion, demonstrating that high loads can cause annulus fibrosus failures or endplates junction failures (AFF and EPJF, respectively), especially when the discs were degenerated or already injured [1,2,3]. In recent years, combined loads and the increase of the intradiscal pressure were considered the main causes of disc failure. In particular, it has been demonstrated that flexion combined with axial rotation or compression led to EPJFs and AFFs, and a high nucleus pulposus pressure was unsafe for the disc [4,5,6].

Despite the many experimental studies presented, it is still unknown which mechanical condition is mostly responsible for the disc failure. The reason is that experimental studies are not directly comparable because of the different species and the different experimental conditions used during the experimental tests [4,7,8,9,10,11,12,13]. Although the human spine is the gold standard in spinal research, there are many disadvantages related to the use of humans. Firstly, they are characterized by a high degree of variability due to the different age and anatomical features of the donors, which causes some difficulties in comparing results. Moreover, human specimens are expensive and not easy to gather in high numbers. For this reason, many biomechanical studies have been performed to investigate which animal model is the most suitable for the human spine, showing that for the lumbar segment, ovine is the most adequate model of human [14,15,16,17,18,19,20,21,22].

Finite element analysis partly overcomes the problems related to experimental testing, and in our opinion, it is an essential tool to understand which mechanical conditions may induce disc failures. It also allows to explore where the maximum stresses and strains are located and their entity, and their dependence by the degree of degeneration [22,23,24,25,26].

However, finite element models mostly describe the human disc, therefore they cannot be used to investigate the experimental tests on animals [22,24,25,26,27,28]. To authors’ knowledge, few models of the ovine lumbar disc have been described in the literature. Schmidt and Reitmaier presented a model of the lumbar ovine disc, which had some limitations related to its anatomical characteristics and the material properties assigned [22]. Reutlinger and colleagues [29] developed an anisotropic model of the ovine lumbar disc, but it has been validated using human data and they did not perform any experimental test to investigate the mechanical failure.

The aim of this study was to develop a criterion for predicting the risk of failure in complex loading conditions. A finite element model of the ovine disc with anisotropic hyperelastic properties [30] was used to perform numerical simulations conducted in a parallel in vitro study [31] on ovine lumbar segments. A statistical investigation based on the experimental and numerical outcomes was performed, leading to estimate the stress condition responsible for the failure of the tissue.

## 2. Materials and Methods

A previously developed FE model of the ovine lumbar intervertebral disc was used for this study. Details of the model have been described elsewhere [30] and are briefly summarized here. The geometry of the model was based on the reconstruction of the endplates (EPs) of the cranial and caudal vertebrae of a L3–4 segment, from which the disc was generated using a custom Python script. The segment was supposed to be representative of the lumbar spine. The disc model was composed by the annulus fibrosus (AF), the nucleus pulposus (NP), the bony and the cartilagineous EPs (BEPs and CEPs, respectively). The anterior and posterior height of the AF was 4.5 mm and 2.5 mm, respectively. The width and the depth were 30 and 22 mm. The CEPs and the BEPs had a thickness of 0.1 and 0.5 mm. The AF was divided in the anterior, lateral and posterior regions. An anisotropic hyperelastic formulation [28] was assigned to the AF and the parameters were determined by a full factorial optimization method. The mechanical parameters of the other structures were taken from the literature (Table 1). The initial NP pressure was set to 0.2 MPa by a specific VUMAT subroutine that simulated the nucleus swelling.

The mesh was composed by 56,496 hexahedral elements and 60,145 nodes. A mesh convergence analysis was performed to ensure the reliability of the model, and finally the model was validated comparing its flexibility with the data presented by Reitmaier and colleagues [15], showing a good agreement of the results [30].

The boundaries and the loading conditions applied by Berger-Roscher et al. [31] were replicated in the simulations. In the experimental study, thirty ovine lumbar segments were collected and the posterior elements were carefully removed. The cranial and the caudal vertebral bodies were embedded and five different loading scenarios applied (Table 2).

In addition, the finite element model was constrained at the caudal EP in all degrees of freedom, and an application node was defined and coupled to the superior EP, allowing the application of rotations in all directions. Five different loading scenarios were simulated in quasi-static conditions (Table 2); the loads applied replicated the conditions described in the experimental study conducted by Berger-Roscher et al. [31], which obtained AFFs and EPJFs. The loading angles were chosen to be double of the values measured by Reitmaier et al. [15] applying a moment of 3.75 Nm without the posterior elements. A compressive load of 800 N has been demonstrated to correspond to the load on the ovine IVD during regular activities (e.g., standing up and lying down) [33].

**Figure 1:**
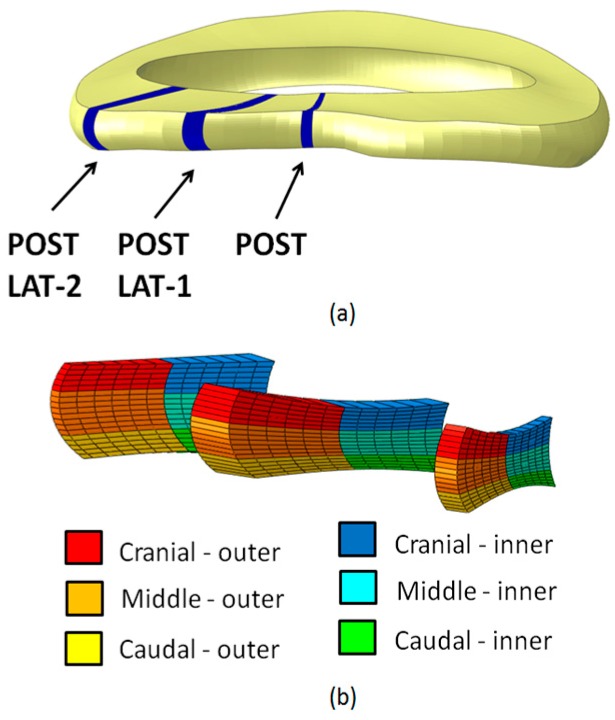
(**a**) Section of the annulus fibrosus in which the stress was analyzed; (**b**) division in subsections of the sections represented in (**a**).

The simulations were performed in Abaqus Explicit 6.12-3 (Simulia, Dassault Systemes, Providence, RI, USA). In order to fulfill the requirement of the quasi-static conditions, it was checked that during the simulations the kinetic energy was lower than 10% of the total energy. Because the aim of the study was to investigate which state of stress was responsible for the failure of the AF and of the EP, the stresses in the circumferential, axial and radial direction, and the stress at the interface between the AF and the EPs were analyzed. In particular, three sections of the AF were defined and each section was divided in six subsections (Figure 1).

The stress was spatially averaged over the inner and outer, and cranial, middle and caudal AF. The stress at the interface between the AF and the EP was calculated as the ratio between the nodal forces and the cross-sectional area.

Multiple linear regression analysis was performed to investigate the role of each stress in predicting the failure of the disc.

The statistical analysis for the comparison of the groups was based on a score that described the damage of the disc. As described by Berger-Roscher and colleagues, small and large EPJFs were distinguished. Furthermore, the AFFs were distinguished in large and small. The evaluation was based on video and micro-CT and MR images of the experimental tests. A score of 0.5 was assigned when small failures occurred, whereas 1 was assigned when large failures occurred. The score was calculated for each specimen using the formula:

(1) S=LargeEPJF+SmallEPJF+LargeAFF+SmallAFF

A vector containing the values of the experimental results was defined, whereas the predicted stresses were inserted in a matrix in which each row was referred to a specimen and the statistical investigation was performed. The outputs of the analysis were the stresses that had a significant influence in predicting the failure of the disc.

Additional FE simulations were performed to better understand the influence of each load in generating an unsafe stress in the disc. The same loads were applied combined in couples or alone (Table 3). The average stress was calculated in the regions in which the stress resulted significant in the previous analysis and compared with the complex loading conditions.

## 3. Results

The numerical results were in good agreement with the experimental ones [31] and allowed defining a criterion to predict the failure of the disc. A statistical analysis allowed establishing which stresses had an influence in predicting AF and EP damaging.

### 3.1. Complex Loads

The numerical outcomes showed that the application of rotations in all main-planes (case 1 and 2) generated the highest stress state, especially in the postero-lateral and anterior regions in the axial and circumferential direction. In fact, since the AF was considered as a continuum material, in the posterior region where the AF is stretched, the applied loads generated a tensile state in the axial direction and a compressive state in the circumferential one. In contrast, in the anterior part where the annulus was compressed, it was subject to a compressive state in the axial direction and a tensile state in the circumferential one.

**Figure 2:**
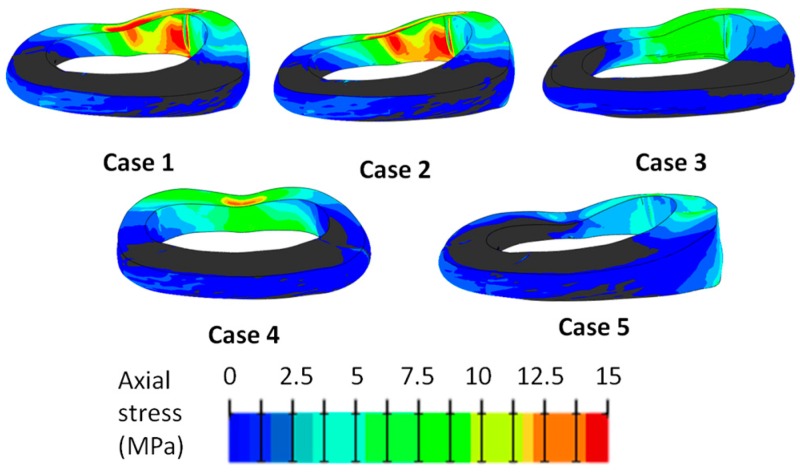
Tensile axial stress generated in the annulus fibrosus in the loading cases listed in Table 2. The stress is expressed in MPa. Areas with negative stresses are shown in gray.

**Figure 3:**
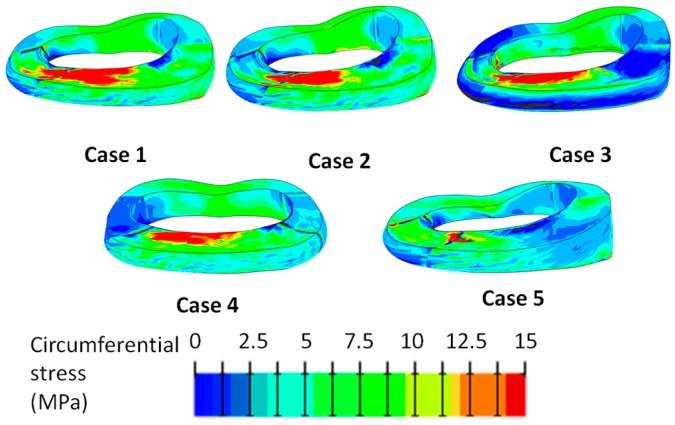
Tensile circumferential stress generated in the annulus fibrosus in the loading cases listed in Table 2. The stress is expressed in MPa. Areas with negative stresses are shown in gray.

Removing the axial compression did not change the stress distribution in the AF, whereas it appeared lower when lateral bending or flexion was not applied (case 4 and 5, respectively). This result demonstrated that the applied moments had a higher influence than the pure compression on the stress state within the AF. In axial direction, the highest tensile stress was located in the inner AF of postero-lateral part (Figure 2), whereas in the circumferential direction it was higher in the anterior region (Figure 3). In the radial direction the stress was the lowest, and the peak was located in the outer caudal region of the postero-lateral AF. The stress distribution did not appear uniform within the AF; in the posterior region, the axial stress was higher in the middle than in the cranial and caudal regions, whereas in the circumferential direction it was higher close to the EPs.

The statistical analysis revealed that the axial and the circumferential stresses were significant to predict the failure of the disc. Because of the non-homogeneous distribution of the stress along the AF, the stress values in three different posterior and postero-lateral regions (POST, POST-LAT1 and POST-LAT2) in which the failure experimentally occurred were calculated and averaged. The most predictive stresses were found in the caudal and in the middle part of the AF, and in the inner region (Table 4).

In the in vitro experiments, the highest level of damage was observed for the loading cases 1 and 2, whereas no damage was found for group 5 (Table 5). In particular, the application of high lateral bending and flexion had a strong influence in generating AFFs (loading cases 1–3) and the combination of all loads causes EPJFs.

**Figure 4:**
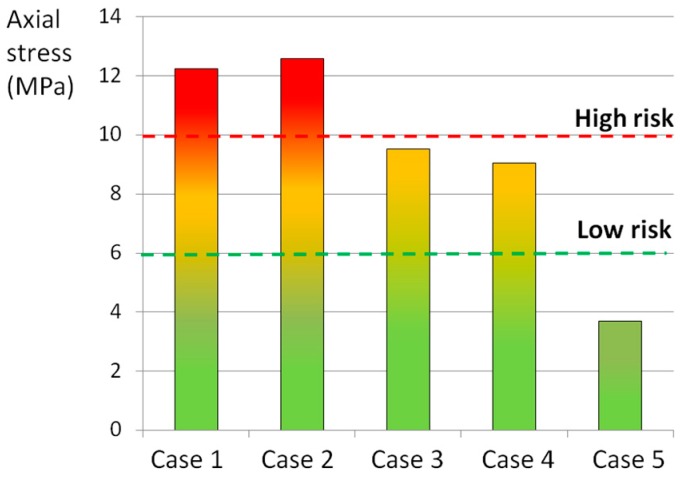
Average tensile axial stress generated by the loading cases listed in Table 1 in POST-LAT1 section. The red and the green lines indicated the thresholds identified as “high risk” and “low risk” of failure, respectively.

**Figure 5:**
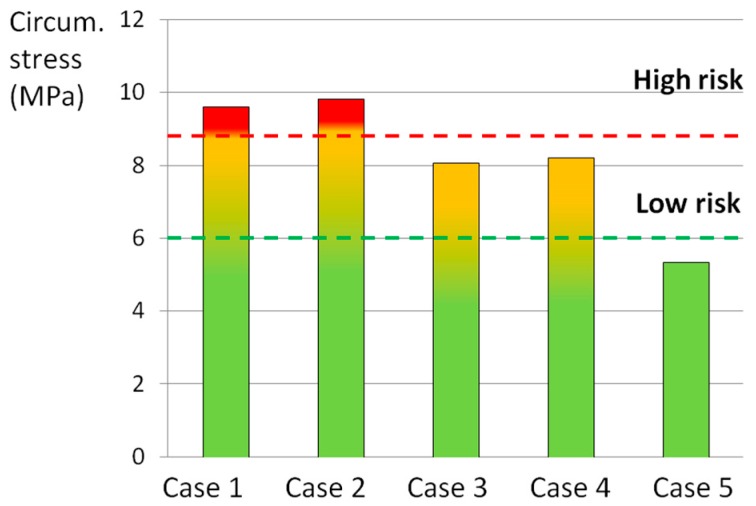
Average tensile circumferential (Circum.) stress generated by the loading cases listed in Table 1 in POST-LAT1 section. The red and the green lines indicated the thresholds identified as “high risk” and “low risk” of failure, respectively.

**Figure 6:**
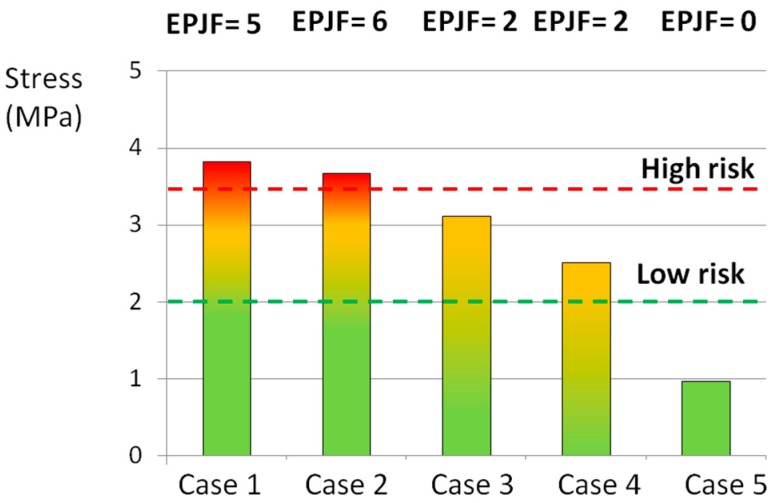
Average tensile stress generated by the loading cases listed in Table 1 at the interface between the annulus and the caudal endplate in the POST LAT-2 section. The red and the green lines indicated the thresholds identified as “high risk” and “low risk” of failure, respectively.

The statistical analysis revealed that the axial and the circumferential stress, as well as the stress at the interface between the AF and the caudal EP, were predictive of the experimental outcomes (Figure 4, Figure 5 and Figure 6). In fact, the comparison between the experimental (Table 5) and the numerical results showed that an axial stress of 12 MPa can generate the disc failure (loading cases 1 and 2), whereas an axial stress of 4 MPa does not generate any damage (case 5) (Figure 4).

In the circumferential direction, a stress of 10 MPa can generate the disc failure (loading cases 1 and 2), whereas an axial stress of 5 MPa does not generate any damage (case 5) (Figure 5).

An axial stress of 10 MPa was qualitatively identified as high risk for the AF failure, whereas the limit of 6 MPa was identified as the threshold defining a low risk of failure (Figure 4). In the circumferential direction the thresholds were lower (9 and 6 MPa for the high and low risk of failure, respectively) (Figure 5). By means of the numerical analysis, it was concluded that a stress higher 3.5 MPa at the interface between the AF and the EP was responsible for the disc damage (Figure 6).

### 3.2. Simple Loads

**Figure 7:**
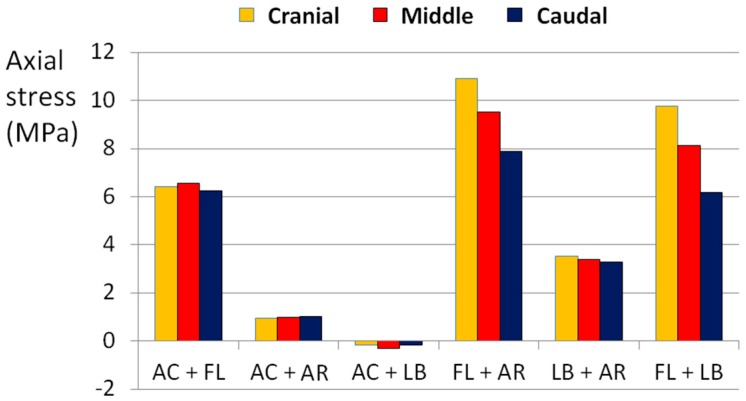
Axial stress generated in the POST section by combined loads. AC means axial compression, FL means flexion, AR means axial torsionrotation, LB means lateral bending.

**Figure 8:**
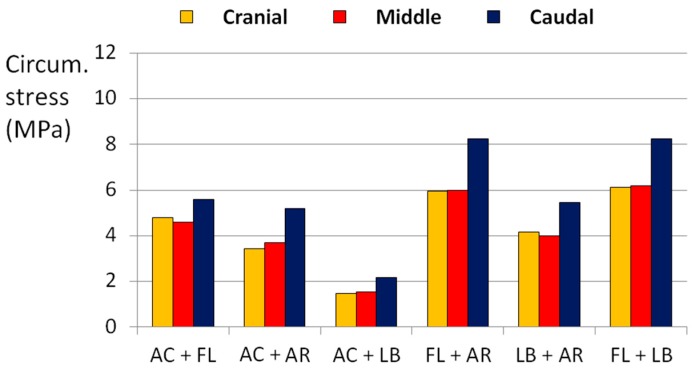
Circumferential (Circum.) stress generated in the POST-LAT1 section by combined loads. AC means axial compression, FL means flexion, AR means axial torsionrotation, LB means lateral bending.

The application of flexion with other loads always generated the highest state of stress in all directions. In particular, the combination of flexion and axial rotation generated an axial stress higher than 10 MPa (Figure 7), whereas flexion together with lateral bending or axial torsion generated a circumferential stress higher than 8 MPa (Figure 8).

**Figure 9:**
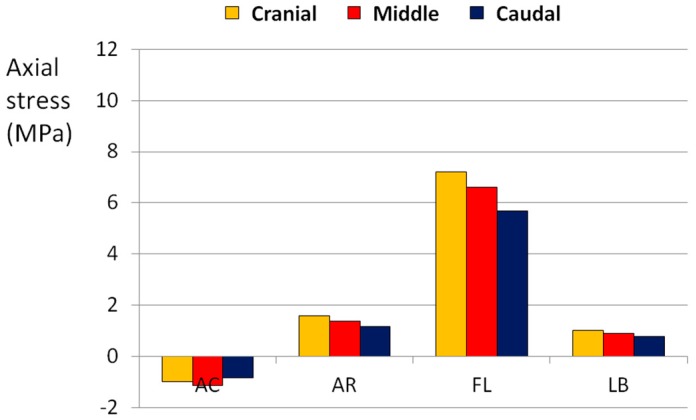
Axial stress generated in the POST section by pure loads. AC means axial compression, FL means flexion, AR means axial torsionrotation, LB means lateral bending.

**Figure 10:**
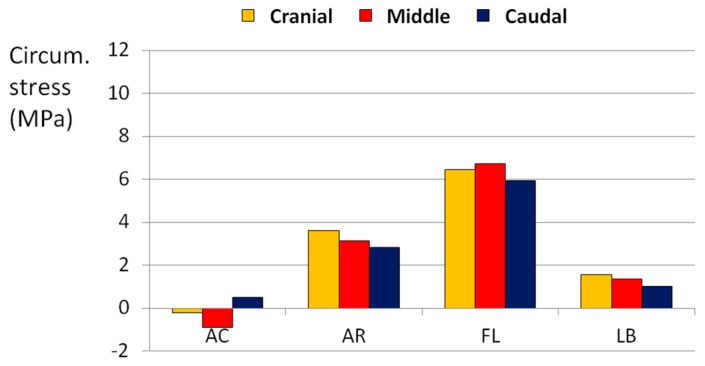
Circumferential (Circum.) stress generated in the POST section by pure loads. AC means axial compression, FL means flexion, AR means axial torsionrotation, LB means lateral bending.

The analysis of the application of single rotations or compression only demonstrated that flexion generated an axial stress up to 7 MPa (Figure 9) and a circumferential one up to 6 MPa (Figure 10).

The thresholds identified for the high and low risk of failure were qualitatively defined on the basis of the comparison with the experimental results. In cases 1 and 2, in which the highest total scores were reached (Table 5), the axial stress was up to 12 MPa, whereas in case 4, in which the damage was localized in posterior part of the AF, the stress reached 10 MPa. When flexion was removed (case 5), no failures occurred and the stress state never reached 6 MPa. Finally, axial rotation seemed to have a moderate influence on the generation of EPJFs: in the in vitro study half of the specimens presented EPJFs and the axial stress was between 6 and 10 MPa.

If only the EPJFs occurrence was considered, the application of all moments seemed to be influent. In fact, in the first two groups there was a high occurrence of failures and a stress state up to 3 MPa. No EPJFs were generated in Group 5, in which the stress at the interface was 1 MPa, whereas in Group 3 and 4 the risk was moderate.

## 4. Discussion

A finite element investigation of the risk of failure of the intervertebral disc was performed. The analysis was conducted in parallel with an in vitro study in which five different complex loading scenarios were applied to generate AFFs and EPJFs. The numerical outcomes confirmed the experimental results, and their combination allowed defining thresholds of the state of stress that identified the risk of generating failures. As well as the experimental tests demonstrated that the combination of the loads in all directions generated failures in the postero-lateral annulus, the numerical simulations showed that the stress was highest in the postero-lateral region. The numerical analysis showed that the combination of all loads (case 1) generated the highest stress values. Axial compression did not increase the state of stress in the AF, probably because the axial load was supported by the NP. In contrast, lateral bending seemed having a great influence on the axial stress, whereas axial rotation increased the circumferential one. In fact, when lateral bending was not applied, the positive stress in the axial direction was lower, whereas when axial rotation was not applied the positive stress was lower in the horizontal direction. The absence of flexion caused the decrease of the tensile stress state in the posterior annulus in both the axial and the circumferential directions.

Flexion was considered as the main load responsible for the disc failure. Indeed, when it was combined with lateral bending or with axial rotation only, or when it was applied as a pure load, the predicted stresses were higher than the threshold values determining a high risk of failure. In contrast, the application of complex loading scenarios without flexion kept the AF in a low risk of failure, although the loads were the double of the physiological ones. The major influence of flexion was demonstrated by the fact that it generated stress up to 7 MPa by itself, whereas with the other loads the stress was lower than 4 MPa in all directions (Figure 9).

In all cases, the tensile stress values were highest in the axial and in the circumferential directions, which defined the plane in which the collagen fibers lay. For the sake of simplicity, the stress distribution was analyzed only where failures experimentally occurred and not in the other regions of the AF. In the posterior and in the postero-lateral regions, the tensile stress was highest in the axial direction than in the circumferential one, whereas in the anterior region, which was in a compressive state, the stress was highest in the circumferential direction.

The numerical results were compared with the experimental responses of the disc under the same loading conditions, giving a better understanding of the mechanical causes of the AF damage. Many numerical investigations [25,26,27,34,35,36] showed that the highest stresses and strains were located in the posterior and in the postero-lateral region of the AF. O’Connell et al. showed by an MRI based set-up that in the human discs the strains are the highest in the posterior region of the annulus in all loading conditions [37]. Qasim and colleagues [26,36] developed a model of the human lumbar disc to predict the damage evolution. They showed that in the healthy disc, the damage started in the posterior region of the AF, close to the inferior EP, and then progressed in the postero-lateral region. In accordance to our study, they showed that when pure loads were applied, flexion generated the highest stress state in the AF and in the EPs, identifying flexion as the main responsible for the failure, whereas axial rotation and lateral bending had a lower effect. In contrast to our model, they reported that in a complex scenario, axial compression reduced the number of cycles to failure. This difference could be due to the method that they used to describe the damage evolution, which was based on the mechanical response of the matrix and not of the collagen fibers. Schmidt et al. [25] investigated the effect of combined moments together with compression on the human lumbar disc. The study demonstrated that the collagen fibers had the highest strain in the postero-lateral region, and in general, the strain was higher when combined loads were applied. However, the authors did not simulate damage or failure.

In this study, an investigation on the failure of the lumbar intervertebral disc of the sheep has been presented. It has been demonstrated that the ovine disc is a good model of the human lumbar one [20,21], and it has been adopted in many biomechanical studies [13,14,15,16,33,38,39,40,41]. Schmidt and Reitmaier [22] investigated the differences between the human and the ovine lumbar disc, concluding that despite the large geometrical differences they are adapted to produce similar internal stresses. A finite element model that can support an experimental protocol has been here presented, and it can predict the safety or the risk of failure of the disc in all loading conditions. The implementation of this study on human specimens has some issues: by an experimental point of view, the main problems are related to the large variability and to the lower bone density [21], which could cause some experimental problems as the vertebral failure. As a consequence, different material properties should be assigned to the numerical model according to the level of degeneration of the subjects. Recently, some investigation have been performed to get a subject-specific modeling of the IVD [42], but the inclusion of the anatomical, mechanical and degenerative properties of the disc into a unique model is a big issue [43]. Long and colleagues [43] have defined some design requirements for the repair hydrogels for human disc, but no information are available for the ovine one, despite it is often used in research [44,45,46,47,48,49,50].

This study has some limitations. First, the FE model used for this investigation did not take into account the viscoelastic and poroelastic properties of the IVD, although many FE models of the human IVD have been developed [48,49,50,51,52,53,54]. However, the poroelastic parameters of the ovine disc are not available and only the failure properties of the tissue were investigated, therefore a hyperelastic model was preferred. Second, the radial fibers were not included in the model. Reutlinger et al. [29] showed that in the ovine lumbar disc the inclusion of the interlamellar fibers changed the state of stress in the disc but did not affect his behavior in terms of displacements. Third, the simulation was performed on a model that was representative of a L3–4 disc, whereas the experiments included the whole lumbar spine. Because the geometrical features have a main role in the mechanical response of the IVD [22], further investigations including anatomical differences should be done.

In fact, the presented model did not aim to predict the exact stress values generated within the AF: the IVD failure is a complex phenomenon that depends by biological [51,52,53,54,55], pathological [5,6,14,56,57,58,59] and mechanical factors [2,3,4,5,6,7,8,9,10,11,12,13,14,31,60,61,62] that cannot be included in the same numerical model. Despite this, the combination of experimental [31] and numerical results on the basis of a statistical investigation allowed identifying if a stress was predictive or not of the failure in a specific region. The conclusions were not based on a pre-defined limit of failure but on the predictivity of the stress values relative to the experimental outcomes.

## 5. Conclusions

A numerical investigation of the state of stress generated by complex loading conditions and responsible for the failure of the AF was presented. The combination of the numerical results with a parallel in vitro study allowed understanding which stress condition was responsible for the disc failure. It was concluded that a tensile axial stress higher than 10 MPa and a positive circumferential stress higher than 8 MPa can generate the failure of the AF, and that flexion is the load that leads the disc to the most unsafe condition. The model can predict the risk of failure in every other loading condition, as well as in models of the entire motion segments or including implantable devices.

## Figures and Tables

**Table 1:** Material behavior of the components of the intervertebral disc (IVD).

Structure	Material Behavior	C_10_ (MPa), D (MPa^−1^)	K_1_ (MPa), K_2_ (MPa), κ	References
Anterior AF	Anisotropic hyperelastic	0.06046, 0.311	24, 1700, 0.01	[30]
Lateral AF	Anisotropic hyperelastic	0.0327, 0.6154	5, 940, 0.01	[30]
Posterior AF	Anisotropic hyperelastic	0.0772, 0.2609	1, 50, 0.01	[30]
NP	Neo-Hookean	0.16779, 0.12	-	[32]
		**E (MPa), υ**		
CEP	Linear elastic	24, 0.4	-	[32]
BEP	Linear elastic	1000, 0.3	-	[32]

AF means annulus fibrosus, NP means nucleus pulposus, CEP means cartilaginous endplate, BEP means bony endplate.

**Table 2:** Loading scenarios investigated in the numerical simulations.

Loading Scenario ^1^ (Case No.)	Axial Compression (800 N)	Axial Rotation (4°)	Lateral Bending (10°)	Flexion (13°)
1	X	X	X	X
2	-	X	X	X
3	X	-	X	X
4	X	X	-	X
5	X	X	X	-

^1^ The loading scenarios corresponded to the experimental groups investigated by Berger-Roscher and colleagues [31].

**Table 3:** Simple loading scenarios investigated in the numerical simulations.

Loading Scenario	Axial Compression (800 N)	Axial Rotation (4°)	Lateral Bending (10°)	Flexion (13°)
AC + FL	X	-	-	X
AC + AR	-	X	-	-
AC + LB	X	-	X	-
FL + AR	-	X	-	X
LB + AR	-	X	X	-
FL + LB	-	-	X	X
AC	X	-	-	-
AR	-	X	-	-
FL	-	-	-	X
LB	-	-	X	-

**Table 4:** Significance of the state of stress in predicting the damaging of the IVD in different locations. The stress responsible for the EPJFs was calculated only for POST-LAT2 (*p*-values < 0.05 are marked with *).

Section	Subsection	Axial	Circumferential	Radial	EP
POST	Cranial	-	-	-	-
	Middle	*	*	*	-
	Caudal	*	*	-	-
	Inner	*	*	*	-
	Outer	-	*	-	-
POST LAT-1	Cranial	-	-	-	-
	Middle	*	*	-	-
	Caudal	*	*	-	-
	Inner	*	*	-	-
	Outer	-	-	-	-
POST LAT-2	Cranial	*	-	*	-
	Middle	*	-	*	-
	Caudal	*	*	-	*
	Inner	*	*	-	-
	Outer	*	-	-	-

**Table 5:** Annulus and endplate junction failures obtained in the in vitro test.

Loading Scenario (Case No.)	Large AFF	Small AFF	Large EPJF	Small EPJF	Total Score ^1^
1	6	0	6	0	12
2	4	0	3	3	8.5
3	4	1	3	0	7.5
4	1	4	1	1	4.5
5	0	0	0	0	0

^1^ The sum of the scores describing the level of damage within each experimental group is reported on the basis of macroscopic, micro-CT and MR images evaluation [31].
